# Effectiveness of the spirometry-based motivational intervention to quit smoking: RESET randomised trial

**DOI:** 10.1080/13814788.2023.2276764

**Published:** 2023-11-07

**Authors:** Francisco Martín-Luján, Antoni Santigosa-Ayala, Meritxell Pallejà-Millán, Cristina Rey-Reñones, Felipe Villalobos, Rosa Solà, the researchers of the RESET study

**Affiliations:** a Department of Primary Care Camp de Tarragona, Institut Català de la Salut, Tarragona, Spain; b CENIT Research Group, Institut Universitari d’Investigació en Atenció Primària Jordi Gol (IDIAP JGol), Barcelona, Spain; c Primary Healthcare Research Support Unit Camp de Tarragona, Institut Universitari d’Investigació en Atenció Primària Jordi Gol (IDIAP Jordi Gol), Reus, Spain; d School of Medicine and Health Sciences, Universitat Rovira i Virgili, Reus, Spain

**Keywords:** Smoking cessation, public health, motivational interventions, spirometry, primary healthcare

## Abstract

**Background:**

The effectiveness of providing feedback on spirometry results for smoking cessation remains inconclusive according to the current evidence.

**Objectives:**

To assess the effectiveness of a motivational intervention based on spirometry results in achieving prolonged smoking abstinence (12 months post-intervention).

**Design and Setting:**

A randomised, controlled, observer-blinded, multicentre clinical trial was conducted (from January 2012 to December 2015) in 20 primary healthcare centres in the Tarragona province, Spain.

**Methods:**

Participants, active smokers aged 35–70 without known respiratory disease, were recruited from primary healthcare centres by family doctors and nurses. They were randomly assigned to either the intervention group (IG = 308) or the control group (CG = 306). Both groups received brief smoking cessation counselling. Additionally, the IG underwent spirometry and received detailed information about the results, including lung age. The primary outcome was prolonged abstinence, defined as lasting at least 12 months and validated through cotinine measurement in urine.

**Results:**

The prolonged abstinence rate was 7.8% in the IG, compared to 2.6% in the CG (*p* = 0.004). At 12 months, in the multivariate analysis, the intervention was identified as an independent factor for smoking cessation (*OR* 2.8; 95%*CI* 1.2 to 7.7), a trend maintained throughout the follow-up (*HR* 2.74; 95%*CI* 1.13 to 6.62). Moreover, according to the Prochaska and DiClemente model, the preparation or action phase to quit was also associated with smoking cessation (*HR* 2.55, 95%*CI* 1.07 to 6.09).

**Conclusion:**

A primary care-delivered intervention involving brief counselling and detailed spirometry information proves effective in increasing abstinence rates among active smokers without known respiratory disease. Additionally, smoking cessation is also influenced by the individual’s stage of change.

**Trial Registration:**

ClinicatTrials.gov NCT02153047

## Introduction

KEY MESSAGES

- In this study, primary care delivered intervention, based on brief counselling plus information on pulmonary function measured through spirometry, almost triples prolonged abstinence rates compared to brief counselling alone.
- In addition to the intervention delivered, another significant factor associated with achieving prolonged abstinence was an advanced stage of the change process.
- Reporting spirometry results could be added as a strategy to enhance smoking cessation efforts.

Despite the well-known deleterious effects of tobacco, smoking rates do not decrease. This might be explained by the addictive nature of tobacco and the underestimation of risks among smokers [1]. Considerable progress has been achieved in the field of tobacco control to reduce its prevalence [2–4]. Nevertheless, the percentage of smokers attempting to quit and maintaining abstinence without assistance remains low (3–5% per year). Some smoking cessation interventions aimed at increasing motivation can be successful in specific contexts [5]. In an earlier study by Parkes et al. an intervention explaining the biological effects of smoking on the body through spirometry results in improved abstinence rates [6]. However, a recent systematic review, including the impact of explaining spirometry results on abstinence rates, concluded that further research is needed before issuing evidence-based recommendations [7].

In a previous study conducted by our group, we reported that brief advice, combined with detailed information on spirometry, doubled rates of prolonged abstinence compared to short advice alone in smokers without known respiratory disease at the 12-month follow-up [8]. In that study, all participants underwent spirometry and received brief advice; however, only the intervention group received detailed information about the spirometry result, while the control group was informed solely about the normality or otherwise of the test. Although the way patients were informed of the results was different, the role of spirometry compared to no spirometry could not be evaluated [9]. Therefore, in the present study, we will assess whether an intervention providing brief advice plus explaining spirometry results to patients can lead to higher rates of behaviour change and smoking cessation compared to giving brief advice alone.

## Methods

The RESET study (REsults, Spirometry, Effectiveness and Tobacco) is a randomised, controlled, observer-blinded, multicentre clinical trial conducted in the primary care setting involving active smokers with no history of respiratory disease. This study constitutes the second phase of the previous ESPITAP study [8]. It aims to assess the effectiveness of usual smoking cessation counselling compared with smoking cessation counselling plus information about the participant’s spirometry results. The study has been registered on the ClinicalTrials website (NCT02153047) and its protocol has already been published [10].

### Participants

The participants were selected at the screening visit (V0) by the referring family doctors and nurses among patients attending their primary healthcare centres for any reason between August 2012 and December 2013 from the population assigned to 20 Catalan Institute of Health primary healthcare centres (12 urban and eight rural) in Tarragona province (Catalonia, Spain).

Inclusion criteria: Active smokers aged 35–70 years, with a cumulative consumption > 10 packs-year. Exclusion criteria: History of respiratory diseases, spirometry performed within the previous 12 months, or inability to follow-up the study protocol.

### Intervention

At the screening visit (V0), all eligible participants were informed of the health risks associated with smoking and advised to quit. In addition, they were informed about the study and allowed to participate. Subjects who met inclusion/exclusion criteria and accepted received a letter of invitation, an informed consent form, and an information sheet providing a detailed explanation of the study. After signing the informed consent, participants were randomised and assigned to either the intervention group (IG) or the control group (CG) in a 1:1 ratio. The group assignment was observer-blinded, consecutive, and centralised at the IDIAP Jordi Gol - Tarragona Research Support Unit, following a simple randomisation numeric sequence compiled for this purpose.

During the inclusion visit (V1), the following information outlined in the study protocol was collected [10]: clinical and demographic data, smoking habits, the stage of change according to Prochaska’s and DiClemente’s model and previous attempts to quit smoking [11].

All participants received health education and brief counselling about smoking cessation following the 5 A strategy administered by their referring healthcare professional [12]. Additionally, spirometry was performed on each IG participant following the American Thoracic Society-European Respiratory Society recommendations [13]. The test was conducted by selected nursing personnel with the appropriate technical skills accredited by the Health Studies Institute of the Catalan Government. Participants received standardised information about their spirometry results in a personalised visit lasting about 15 min, explaining the content of the report in detail. The commentary on each spirometry test was prepared from a consensus interpretation by the research team and focused on a structured description of the results obtained and their interpretation concerning a theoretical normal value. Participants were also informed about their lung age [14]. Table 1 describes the characteristics of the intervention.

**Table 1.** Smoking cessation counselling.

Control group Brief smoking cessation intervention (CONTROL Intervention)	Intervention group Brief smoking cessation plus spirometry report (RESET intervention)
The health professional will apply the 5A’s strategy, which includes five steps: Ask, Advise, Assess, Assist, and Arrange	
During a 5-minute intervention, the healthcare professional will provide a clear, personalised recommendation for smoking cessation.	For a 15-minute intervention, the healthcare professional will conduct an intervention with the same content as brief smoking cessation counselling. Additionally, it will provide information about the spirometry results and address any questions related to spirometry or other issues.
For A2 (advice): They will explain to the smoker that the most impactful decision for improving their health is to quit smoking and will provide written informational materials outlining the benefits of smoking cessation. The materials are sourced from the ‘Smoke-Free Primary Care’ programme of the Catalan Society of Family Medicine and the Public Health Agency of Catalonia, regularly employed in primary care for brief smoking cessation interventions.	For A2 (advice): If spirometry values are within normal range, the patient will be informed that their pulmonary function has not yet deteriorated, and that this would be an opportune time to quit smoking. If spirometry values indicate airway obstruction (FEV1/ FVC < 70%), the patient will be informed that they could have chronic obstructive pulmonary disease caused by smoking, and that the most important measure is to quit smoking. If spirometry values show FVC < 80% (airway restriction), the patient will be informed that their pulmonary function could be affected and will be advised to continue with the pulmonary tests normally performed in primary care. Additionally, the patient will be informed about their lung age (i.e. the mean age of a non-smoker with the same FEV1) compared to their chronological age to illustrate the deterioration of the lungs due to smoking.

To illustrate the deterioration of lung function caused by tobacco smoking, all participants were informed using the Fletcher diagram [15]. They were also offered a specific ‘quit smoking’ consultation. Furthermore, patients with abnormal spirometry (Forced Vital Capacity [FVC] < 80%, Forced Expiratory Volume in the first second [FEV1] < 80% and/or FEV1/FVC ratio < 0.7) were informed and referred to their family doctor.

All participants were assessed during several telephone follow-up visits with the referring healthcare professional. After 3–6 months (V2) and 6–9 months (V3) post-inclusion, visits were conducted to provide repeated smoking cessation counselling and to monitor changes in smoking habits. Data about smoking habits were once again collected at the final in-person visit, 12 months post-inclusion (V4).

Each researcher stored all information obtained using an online application accessible exclusively through the Intranet of the Catalan Health Institute in Tarragona (Spain), with password-restricted access.

### Outcomes

Following the recommendations of the Society for Research on Nicotine and Tobacco, the primary outcome was prolonged long-term abstinence after 12 months, confirmed by laboratory testing [16]. This prolonged abstinence was counted from an initial period when smoking is not considered a failure (it is recommended that this period not exceed 2–4 weeks from the intervention) until a 12-month follow-up point. The secondary outcome was prolonged abstinence after six and nine months, and point abstinence (abstinence during a time window immediately before the follow-up point, usually seven days), measured at V4.

All patients who reported smoking cessation had their level of expired-carbon monoxide (CO) determined, and if the value was less than 10 ppm (higher values would indicate smoking in the last 12–24 h) [17], urinary cotinines were measured, with values <100 ng/mL considered abstinent [18]. This urinary cotinine determination was carried out one year after the patient-reported abstinence.

### Sample size

With a final sample of 614 participants, we can detect a ≥ 5% difference between the IG and the CG regarding prolonged abstinence (absolute risk), assuming an abstinence rate of 2.5% in the CG, a power of 80% (beta risk), a significance level (alpha risk) of 5% in a two-tailed contrast, and a lost to follow-up of <5% of patients [8].

### Blinding and statistical methods

The study data were extracted from the centralised database and grouped, ensuring that the researchers responsible for statistical analysis were blinded to study group assignments.

The analysis was based on the intention-to-treat principle (including all participants randomly assigned in V0, regardless of whether they received the allocated intervention and subsequent withdrawal or deviation from the protocol), assuming a ‘worst-case’ strategy regarding smoking (participants who did not have laboratory-confirmed abstinence data at the end of the study were considered as active smokers) [19].

Initially, a bivariate analysis was conducted to assess the comparability and homogeneity of the groups at baseline. The prevalence of prolonged and punctual abstinence after 12 months of the intervention in both groups was compared using the Chi-Square test. Additionally, multivariate logistic regression and Cox regression analyses were performed to identify the independent risk factors associated with abstinence. The results were expressed as Odds Ratios (*OR*) and Hazard Ratios (*HR*), respectively. These analyses included the study’s variable of interest (the intervention), variables that showed statistical significance in the bivariate analysis, and other variables considered of interest: sociodemographic data, physical activity, smoking habits (age of onset, daily consumption, cumulative consumption, addiction, stage of change according to the model of Prochaska and DiClemente), motivation to quit smoking, and previous attempts to quit smoking.

All measures were expressed with their respective 95% confidence interval (*CI*). Statistical significance was set at two-sided *p*-values of 0.05 or less. Analyses and data handling were performed using the R Statistics package (R Foundation for Statistical Computing, Vienna, Austria; version 4.0.5).

## Results

**Figure 1.**
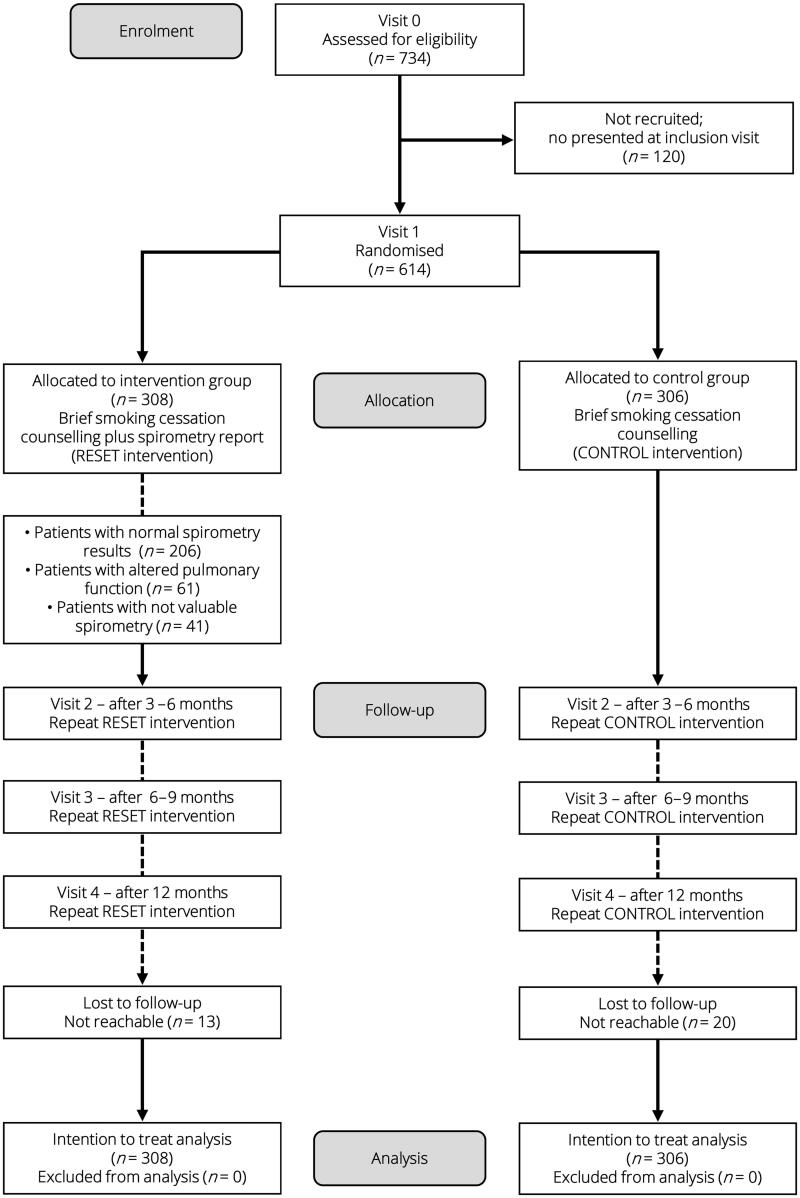
CONSORT (2010) diagram adapted for this study: Screening, randomisation and follow-up of the study participants.

A total of 734 patients were recruited and agreed to participate in the study. Ultimately, 614 were randomised: 308 to the IG and 306 to the CG. Figure 1 shows the CONSORT flowchart.

### Baseline data

Table 2 displays the baseline characteristics of the study population. At baseline, a significant difference between groups was observed only in the distribution of Prochaska and DiClemente stages of change (*p* = 0.03). In the IG, 19.8% (*n* = 61) exhibited some lung function impairment in spirometry. Complete information on baseline data is available in Supplementary Table 1.

**Table 2.** Characteristics of the participants at baseline, according to the randomised assignment group.

	Control *n* = 306	Intervention *n* = 308	p-value*
General information			
Sex (male)	132 (43.1)	146 (47.4)	0.327
Age (years)	51.7 (9.6)	53.1 (9.3)	0.608
Comorbidity			
Hypertension	81 (26.5)	85 (27.6)	0.823
Dyslipidaemia	74 (24.2)	96 (31.2)	0.065
Diabetes mellitus	29 (9.5)	36 (11.7)	0.448
Coronary disease	6 (2.0)	4 (1.3)	0.742
Heart failure	3 (1.0)	1 (0.3)	0.611
Neurological disease	4 (1.3)	4 (1.3)	0.993
Smoking habit			
Cumulative consumption (pack-years)^a^	26.0 (18.1)	27.0 (16.7)	0.462
Daily consumption (cigarettes/day)	13.2 (8.42)	14.02 (8.61)	0.233
Fageström test score	3.9 (2.3)	4.0 (2.4)	0.658
Nicotine dependence^b^			0.743
Low-Moderate	267 (92.7)	274 (91.6)	
High	21 (7.3)	25 (8.4)	
Richmond test score Motivation to quit^c^	5.6 (4.9)	6.1 (3.0)	0.148 0.240
Low-Moderate	168 (58.3)	159 (53.2)	
High	120 (41.7)	140 (46.8)	
Previous attempts			0.931
No	90 (33.5)	93 (32.7)	
Yes	179 (66.5)	191 (67.3)	
Stage of change^d^			0.031
Pre-contemplative	112 (40.1)	96 (32.5)	
Contemplative	137 (49.1)	143 (48.5)	
Preparation/pre-action	30 (10.8)	56 (19.0)	
Pulmonary function			
Normal pulmonary function		206 (66.9)	
Altered pulmonary function (patients)		61 (19.8)	
Not valuable spirometry		41 (13.3)	
% FVC reference		92.7 (17.4)	
% FEV1 reference		95.8 (18.0)	

Data are presented as number of patients (and percentage) or mean (and standard deviation) according to the type of variable.

^(*)^ The *p*-value corresponds to the differences in proportions using the Chi-square test for qualitative variables and the t-Student test for continuous variables.

^a^ Value obtained by multiplying the daily average of cigarettes smoked by the number of years of the habit and dividing by 20.

^b^ Considering dependence: low 0–3, medium 4–7, high 8–10.

^c^ Considering motivation: low 0–3, medium 4–6, high 7–10.

^d^ According to Prochaska’s and DiClemente’s model.

FVC: forced vital capacity; FEV1: forced expiratory volume in 1 s.

### Numbers analysed

At the end of the study, the loss to follow-up was 5.4% (33/614 participants), with no significant differences between groups: 6.5% in the CG (20/306 participants) and 4.2% in the IG (13/308 participants). However, the analysis was based on the intention-to-treat principle and, therefore, included all randomised participants.

### Outcomes

The prevalence of prolonged abstinence was 7.8% (5.29–11.33) in the IG compared to 2.6% (1.33–5.07) in the CG (*p* = 0.004). In the IG, no differences were observed in the prevalence of prolonged abstinence among patients with normal spirometry (17/206) and those with altered spirometry (6/61); (8.3% vs 9.8%; *p* = 0.899).

Adjusted analysis shows that the intervention significantly increased the probability of point and prolonged abstinence during follow-up (at six and nine months), and almost tripled the probability of prolonged abstinence at 12 months (*OR* 2.84; 95%*CI* 1.18 to 7.65). Table 3 presents the data on complete abstinence.

**Table 3.** Observed abstinence rates, unadjusted and multivariable-adjusted odds ratios according to random assignment group (intention-to-treat analysis).

	Quit *n* (%)	Unadjusted OR (95% CI)	Adjusted OR^b^ (95% CI)
Point abstinence			
*Control group*	12 (3.92)	2.64 (1.36 to 5.47)	2.31 (1.10 to 5.13)
*Intervention group*	30 (9.74)		
Prolonged abstinence^a^			
For 6 months			
*Control group*	11 (3.59)	2.79 (1.40 to 5.93)	2.43 (1.13 to 5.57)
*Intervention group*	29 (9.42)		
For 9 months			
*Control group*	9 (2.94)	2.92 (1.38 to 6.71)	2.58 (1.12 to 6.51)
*Intervention group*	25 (8.12)		
For 12 months			
*Control group*	8 (2.61)	3.15 (1.45 to 7.59)	2.84 (1.18 to 7.65)
*Intervention group*	24 (7.79)		

Data are presented as number of patients (and percentage), odds ratio (OR) and 95% confidence interval (CI).

^a^ Considering an initial period of 30 days until the end of the follow-up period at 6, 9 or 12 months from the cessation date and confirmed by urine cotinine values <100 ng/mL at 12 months.

^b^ Logistic Regression analyses adjusted for multiple variables: group (control/intervention), sex (male/ female), age group (≥50/ <50 years), civil status (single or not single), children (yes/ no), social class (according to the classification proposed by the Spanish Society of Epidemiology), physical activity (low/ moderate-intense), smoking onset age (before/after 14 years), smoking cumulative consumption (≥10/ <10 pack-years), nicotine dependence level (low-moderate or high), motivation to quit smoking level (low-moderate or high), previous attempts to quit smoking (yes/no) and stage of change (pre-contemplation, contemplation or preparation-action). Other variables considered but not included in the final model were primary healthcare professional performing the intervention, primary healthcare centre, comorbidity disease, body mass index, alcohol intake categorisation, baseline expired-carbon monoxide values, acceptance of a smoking cessation medical visit, intensive motivational intervention and/or use of pharmacological treatment.

**Figure 2.**
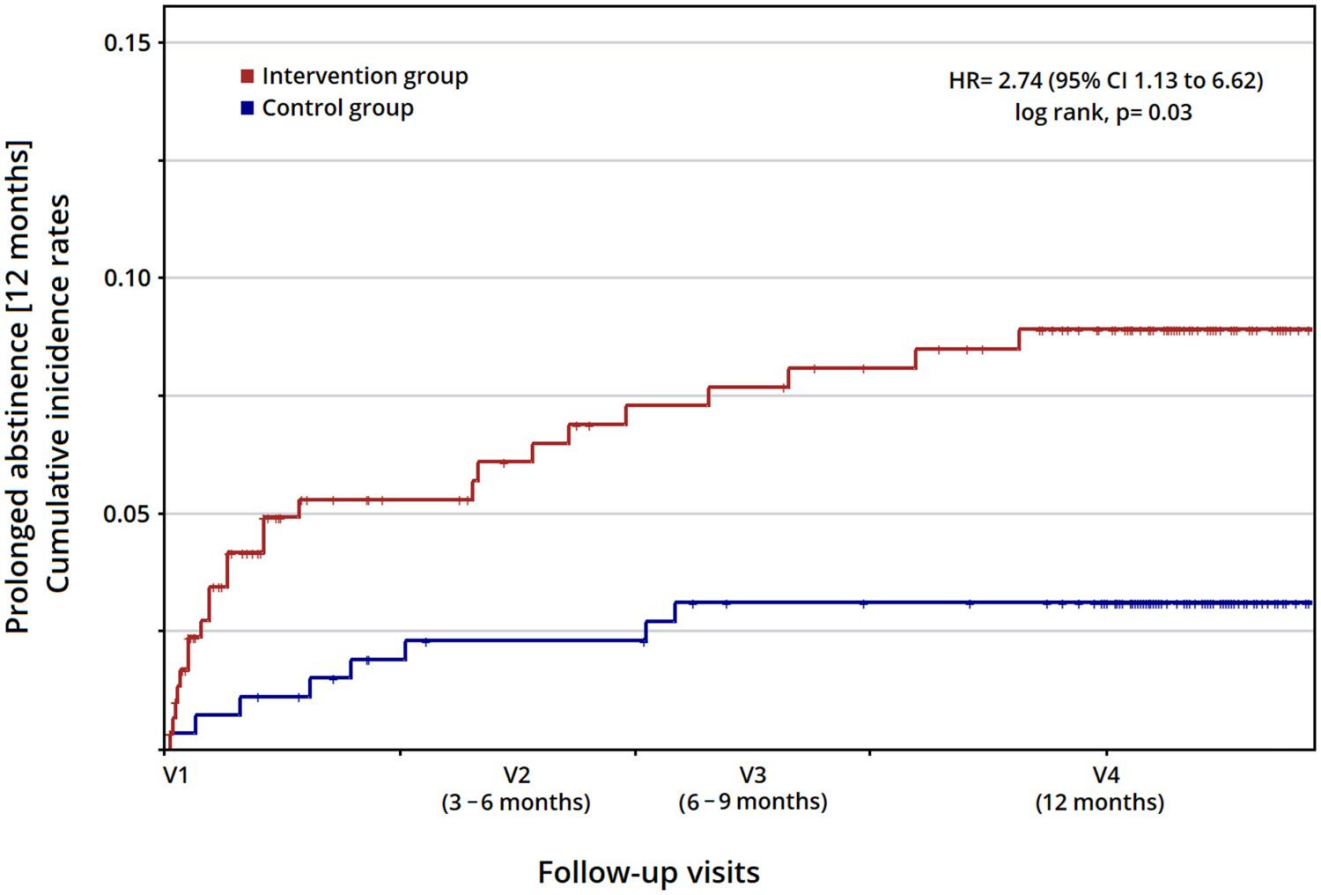
Cumulative incidence rates of smoking cessation during follow-up in the intervention group and control group (analysis from Cox regression models). The figure depicts the cumulative hazard of prolonged abstinence for 12 months. The value of Hazard Ratio (HR) is calculated from Cox regression model adjusted by multiple variables: group (control/intervention), sex (male/female), age group (≥50/<50 years), civil status (single or not-single), children (having/not having), social class (according to the classification proposed by the Spanish Society of Epidemiology), physical activity (low/moderate-intense), smoking onset age (before/after 14 years), smoking cumulative consumption (≥10/<10 pack-years), nicotine dependence level (low-moderate or high), motivation to quit smoking level (low-moderate or high), previous attempts to quit smoking (yes/no) and stage of change (pre-contemplation, contemplation or preparation-action).

Figure 2 illustrates the cumulative abstinence curves between IG and CG, as well as the Cox regression analysis. Throughout the follow-up (V2, V3, and V4), adjusted prolonged abstinence (12 months) was significantly higher in the IG. The factors that independently influenced smoking cessation at 12 months were the intervention (*HR* 2.74; 95%*CI* 1.13 to 6.62) and an advanced stage of change (preparation or action) according to Prochaska’s and DiClemente’s smoking model (*HR* 2.55; 95%*CI* 1.07 to 6.09). The complete results, as well as all adjustment variables, are available in Supplementary Table 2.

## Discussion

### Main findings

The results of the current study confirm the hypothesis that adding information on spirometry results to brief smoking cessation counselling significantly increases the probability of smoking cessation (*OR* 2.8; 95%*CI* 1.2 to 7.7) and maintains it in the long term (*HR* 2.7; 95%CI 1.1 to 6.6). These findings provide new evidence on the effectiveness of spirometry-based smoking cessation strategies in real-world practice.

### Strengths and limitations

This trial constitutes a pragmatic intervention carried out within the usual working conditions of primary healthcare centres, following the 5 A methodology [12]. The 12-month follow-up of participants has allowed us to obtain punctual and prolonged abstinence prevalence (at six, nine and 12 months), as proposed by the Russell standard [20]. Following recommendations, self-reported abstinence was verified using objective biochemical methods (in this study, initially expired CO and subsequently cotinine in urine) [21]. Furthermore, the statistical analysis adhered to the intention-to-treat principle [22]. These methodological considerations enhance the overall study quality, aligning with recommendations in subsequent Cochrane reviews to draw firmer conclusions [23,24].

We also have noted some limitations in our study. First, we highlight that, despite having a large sample size, the originally calculated number of participants outlined in the protocol was not achieved [10]. Nevertheless, since only some participants were lost to follow-up, the obtained sample was sufficient to demonstrate the intervention’s effectiveness. In terms of patient group distribution, randomisation ensured comparability across most variables although we observed statistically significant differences in the distribution of the stage of change according to Prochaska’s and DiClemente’s model. Indeed, in the regression analyses, both variables—study group and stage of change—were identified as the only factors associated with prolonged abstinence. On the other hand, our study exclusively focused on smokers without previous respiratory diseases. Therefore, its results cannot be generalised to people with known respiratory diseases, who may have different motivations and challenges in quitting smoking [25]. In any case, it is essential to note that participants with abnormal medical tests did not exhibit differential responses to the intervention [26]. Finally, comparing brief smoking cessation interventions across other settings could be challenging. Therefore, in this study, we applied the standard 5 A recommendation proposed for primary care [12].

### Comparison with existing literature

Spirometry provides valuable information for the diagnosis of respiratory diseases and has been proposed as an instrument to encourage smokers to quit [23]. However, only a few studies have evaluated its effectiveness, with heterogeneous results.

Some studies conducted in primary care propose a similar objective but present methodological differences, which might explain discordant results. In the first, Parkes et al. concluded that an intervention based on explaining spirometry results, compared to not doing so, was effective in increasing the abstinence rate after 12 months (*OR* 2.29; 95%*CI* 1.28 to 4.13) [6]. However, Parkes et al. did not clearly define the main variable of abstinence (punctual or prolonged abstinence). The abstinence rates achieved (6.4% in the CG and 13.6% in the IG) were closer to the point of abstinence observed in our study (3.92% to in the CG and 9.74% in the IG). The main criticism of Parkes’ study is that the differences between the IG and CG are related more to how the results were explained to the participants (lung age in relation to FEV1 values) than to the performance of spirometry [9].

On the other hand, Kotz et al. evaluated the effectiveness of a more complex and multi-component intervention that included different strategies for smoking cessation [27]. Participants were allocated into three groups and followed up for one year: the IG (*n* = 116) received medium-intensity smoking cessation counselling administered by nurses, along with confrontational advice using spirometry results and a prescription of nortriptyline; the CG-1 (*n* = 112) received medium-intensity smoking cessation counselling and a prescription of nortriptyline; and the CG-2 (*n* = 68) received only low-intensity smoking cessation counselling from a physician. No overall differences between the CG and IG were observed. These methodological differences, especially when evaluating the role of spirometry in combination with other interventions, made it challenging to compare these results with those obtained in our study directly.

The SPIROTAB study evaluated 350 smokers who were initially given spirometry and detailed information about the results as well as brief advice [28]. Follow-up was carried out for two years, and brief advice was repeated at successive visits to all participants. IG participants were given detailed baseline spirometry information again at each visit and retested at the 12-month visit. Reported abstinence was confirmed by cooximetry. Smoking cessation rates at 12 and 24 months were higher in the GI compared to the CG (24% vs. 16.2%, and 25.2% vs 18.4%, respectively), and the overall adjusted odds of quitting smoking in the IG were higher than the CG (OR 1.42; *95%CI* 1.06 to 1.90).

Finally, our research group published the ESPITAP study [8], where all participants (*n* = 571) received brief smoking cessation counselling and underwent baseline spirometry. Still, only the IG was provided with detailed information on the spirometry results and lung age. The main study variable was prolonged abstinence confirmed by expired CO, which at 12 months was 5.6% and 2.1% in the IG and CG, respectively (*p* = 0.04). The cumulative abstinence curves confirmed the better results for the IG (*HR* 1.98; 95%*CI* 1.29 to 3.04). However, the methodology of our previous ESPITAP study has a design that differentiates it from the RESET study. All patients underwent spirometry but only those in the IG received a detailed explanation of the results obtained. At the same time, those in the CG were simply informed of the normality of the test. Thus, it does not allow a comparison of the effectiveness of performing a spirometry test compared to not performing it [29].

In short, while these previous studies allowed for evaluating the potential impact of a spirometry-based intervention to increase tobacco abstinence, our present study is the first to provide data on the independent motivational effectiveness of performing spirometry compared to not doing it.

### Implications for research and practice

The latest Cochrane review evaluates the findings of several trials using spirometry as a motivator for smoking cessation. Its authors conclude that there is insufficient evidence supporting its use. They also recommend improving the methodological quality of studies to yield more robust results [23].

Our results demonstrate that primary care delivered intervention, combining brief counselling with detailed spirometry information, is effective in increasing punctual and prolonged smoking abstinence rates at 12 months. Prolonged abstinence is contingent on the intervention performed and the stage in the change process. These findings contribute new evidence on the effectiveness of health biomarkers feedback for smoking cessation.

### Ethics approval and transparency statement

The study protocol was approved by the Clinical Research Ethics Committee of the IDIAP Jordi Gol (*Institut Universitari d’Investigació en Atenció Primària Jordi Gol;* registration number 4R11/037). The principal investigator guarantees that this study is carried out by national and international legislation and with the principles of the Declaration of Helsinki and the Guidelines for Good Clinical Practice published by the Catalan Institute of Health.

## Supplementary Material

Supplemental Material

Supplemental Material

Supplemental Material

## References

[1] Baker CL, Flores NM, Zou KH, et al. Benefits of quitting smoking on work productivity and activity impairment in the United States, the European Union and China. Int J Clin Pract. 2017;71(1):e12900. doi: 10.1111/ijcp.12900. 28097760 PMC5299499

[2] Bala MM, Strzeszynski L, Topor-Madry R. Mass media interventions for smoking cessation in adults. Cochrane Database Syst Rev. 2017;11(11): CD004704. doi: 10.1002/14651858.CD004704.pub4. 29159862 PMC6486126

[3] McNeill A, Gravely S, Hitchman SC, et al. Tobacco packaging design for reducing tobacco use. Cochrane Database Syst Rev. 2017;4(4):CD011244. doi: 10.1002/14651858.CD011244.pub2. 28447363 PMC6478110

[4] Frazer K, Callinan JE, McHugh J, et al. Legislative smoking bans for reducing harms from secondhand smoke exposure, smoking prevalence and tobacco consumption. Cochrane Database Syst Rev. 2016;2(2):CD005992. doi: 10.1002/14651858.CD005992.pub3. 26842828 PMC6486282

[5] Minian N, Corrin T, Lingam M, et al. Identifying contexts and mechanisms in multiple behavior change interventions affecting smoking cessation success: a rapid realist review. BMC Public Health. 2020;20(1):918. doi: 10.1186/s12889-020-08973-2. 32532233 PMC7291527

[6] Parkes G, Greenhalgh T, Griffin M, et al. Effect on smoking quit rate of telling patients their lung age: the Step2quit randomised controlled trial. BMJ. 2008;336(7644):598–600. doi: 10.1136/bmj.39503.582396.25. 18326503 PMC2267989

[7] Westerdahl E, Engman KO, Arne M, et al. Spirometry to increase smoking cessation rate: a systematic review. Tob Induc Dis. 2019;17:31. doi: 10.18332/tid/106090. 31516474 PMC6662778

[8] Martin-Lujan F, Basora-Gallisa J, Villalobos F, et al. Effectiveness of a motivational intervention based on spirometry results to achieve smoking cessation in primary healthcare patients: randomised, parallel, controlled multicentre study. J Epidemiol Community Health. 2021;75(10):1001–1009. doi: 10.1136/jech-2020-216219. 33883199 PMC8458052

[9] Guirguis-Blake JM, Senger CA, Webber EM, et al. Screening for chronic obstructive pulmonary disease: evidence report and systematic review for the US preventive services task force. JAMA. 2016;315(13):1378–1393. doi: 10.1001/jama.2016.2654. 27046366 PMC13410715

[10] Martin-Lujan F, Santigosa-Ayala A, Piñol-Moreso JL, et al. Multicentric randomized clinical trial to evaluate the long-term effectiveness of a motivational intervention against smoking, based on the information obtained from spirometry in primary care: the RESET study protocol. BMC Fam Pract. 2016;17(1):15. doi: 10.1186/s12875-016-0415-1. 26846522 PMC4743363

[11] Prochaska JO, DiClemente CC. Stages and processes of self-change of smoking: toward an integrative model of change. J Consult Clin Psychol. 1983;51(3):390–395. doi: 10.1037//0022-006x.51.3.390. 6863699

[12] Fiore MC, Baker TB. Clinical practice. Treating smokers in the health care setting. N Engl J Med. 2011;365(13):1222–1231. doi: 10.1056/NEJMcp1101512. 21991895 PMC4494734

[13] Miller MR, Hankinson J, Brusasco V, et al. Standardisation of spirometry. Eur Respir J. 2005;26(2):319–338. doi: 10.1183/09031936.05.00034805. 16055882

[14] Morris JF, Temple W. Spirometric "lung age" estimation for motivating smoking cessation. Prev Med. 1985;14(5):655–662. doi: 10.1016/0091-7435(85)90085-4. 4070195

[15] Fletcher C, Peto R. The natural history of chronic airflow obstruction. Br Med J. 1977;1(6077):1645–1648. doi: 10.1136/bmj.1.6077.1645. 871704 PMC1607732

[16] Hughes JR, Keely JP, Niaura RS, et al. Measures of abstinence in clinical trials: issues and recommendations. Nicotine Tob Res. 2003;5(1):13–26. Erratum in: nicotine Tob Res. 2003;5(4):603. doi: 10.1080/1462220031000070552. 12745503

[17] Sato S, Nishimura K, Koyama H, et al. Optimal cutoff level of breath carbon monoxide for assessing smoking status in patients with asthma and COPD. Chest. 2003;124(5):1749–1754. doi: 10.1378/chest.124.5.1749. 14605044

[18] Raja M, Garg A, Yadav P, et al. Diagnostic methods for detection of cotinine level in tobacco users: a review. J Clin Diagn Res. 2016;10(3):ZE04–6. doi: 10.7860/JCDR/2016/17360.7423. PMC4843405 27135020

[19] West R, Hajek P, Stead L, et al. Outcome criteria in smoking cessation trials: proposal for a common standard. Addiction. 2005;100(3):299–303. doi: 10.1111/j.1360-0443.2004.00995.x. 15733243

[20] Cheung KL, de Ruijter D, Hiligsmann M, et al. Exploring consensus on how to measure smoking cessation. A delphi study. BMC Public Health. 2017;17(1):890. doi: 10.1186/s12889-017-4902-7. 29162043 PMC5696733

[21] Benowitz NL, Bernert JT, Foulds J, et al. Biochemical verification of tobacco use and abstinence: 2019 update. Nicotine Tob Res. 2020;22(7):1086–1097. doi: 10.1093/ntr/ntz132. 31570931 PMC7882145

[22] Detry MA, Lewis RJ. The intention-to-treat principle: how to assess the true effect of choosing a medical treatment. JAMA. 2014;312(1):85–86. doi: 10.1001/jama.2014.7523. 25058221

[23] Clair C, Mueller Y, Livingstone-Banks J, et al. Biomedical risk assessment as an aid for smoking cessation. Cochrane Database Syst Rev. 2019;3(3):CD004705. doi: 10.1002/14651858.CD004705.pub5. 30912847 PMC6434771

[24] Bize R, Burnand B, Mueller Y, et al. Biomedical risk assessment as an aid for smoking cessation. Cochrane Database Syst Rev. 2012;12:CD004705. doi: 10.1002/14651858.CD004705.pub4. 23235615

[25] Kotz D, Wesseling G, Huibers MJ, et al. Efficacy of confronting smokers with airflow limitation for smoking cessation. Eur Respir J. 2009;33(4):754–762. doi: 10.1183/09031936.00116308. 19129277

[26] Ronaldson SJ, Dyson L, Clark L, et al. The impact of lung function case-finding tests on smoking behaviour: a nested randomised trial within a case-finding cohort. Health Sci Rep. 2018;1(6):e41. doi: 10.1002/hsr2.41. 30623078 PMC6266471

[27] Kotz D, Huibers MJ, West RJ, et al. What mediates the effect of confrontational counselling on smoking cessation in smokers with COPD? Patient Educ Couns. 2009;76(1):16–24. doi: 10.1016/j.pec.2008.11.017. 19150590

[28] Rodriguez-Alvarez MDM, Roca-Antonio J, Martínez-González S, et al. Spirometry and smoking cessation in primary care: the ESPIROTAB study, a randomized clinical trial. Int J Environ Res Public Health. 2022;19(21):14557. doi: 10.3390/ijerph192114557. 36361437 PMC9658367

[29] Lin KW. Lung age: study’s conclusion about screening is unwarranted. BMJ. 2008;336(7652):1034–1034. doi: 10.1136/bmj.39556.492176.80. PMC2376028 18467391

