# Supplementary material for: Effectiveness of the spirometry-based motivational intervention to quit smoking: RESET randomised trial
Source: Eur J Gen Pract. 2023 Nov 7;29(1):2276764. doi: 10.1080/13814788.2023.2276764 (PMC10631381; doi:10.1080/13814788.2023.2276764)
Supplement: Supplemental Material [file IGEN_A_2276764_SM1524.docx]

Supplementary Table 2. Cox regression analyses assessing multivariable-adjusted risk factors for abstinence at the 12-month follow-up

|  | **HR** | **95%CI** | **p-value** |
| --- | --- | --- | --- |
| Intervention group | 2.74 | 1.13 to 6.62 | 0.025 |
| Sex (male) | 1.99 | 0.78 to 5.07 | 0.151 |
| Age (≥50 years) | 1.00 | 0.96 to 1.05 | 0.943 |
| Civil status (single or not single) | 1.07 | 0.42 to 2.73 | 0.886 |
| Children (yes) | 2.25 | 0.59 to 8.56 | 0.234 |
| Social Class |  |  |  |
| *Graduates and diploma holders* | 0.57 | 0.09 to 3,81 | 0.563 |
| *Qualified non-manual* | 0.41 | 0.07 to 2.35 | 0.319 |
| *Qualified manual* | 0.53 | 0.09 to 3.04 | 0.472 |
| *Partially qualified* | 1.07 | 0.19 to 6.12 | 0.937 |
| *Unqualified* | 0.30 | 0.05 to 1.90 | 0.202 |
| Physical activity (moderate-intense) | 0.62 | 0.27 to 1.41 | 0.258 |
| Smoking onset age <14 years | 0.41 | 0.12 to 1.42 | 0.158 |
| Cumulative consumption ≥10 pack-years | 0.82 | 0.26 to 2.55 | 0.732 |
| High dependency | 0.49 | 0.06 to 3.83 | 0.496 |
| High motivation | 1.05 | 0.44 to 2.50 | 0.917 |
| Previous attempts to quit smoking (yes) | 1.77 | 0.64 to 4.90 | 0.275 |
| Stage of change (preparation or action) | 2.55 | 1.07 to 6.09 | 0.035 |
| Data are presented as hazard ratio (*HR*), 95% confidence interval (CI) and p-value.  Cox analyses were adjusted for multiple variables: group (control/intervention), sex (male/female), age group (≥50/<50 years), civil status (single or not-single), children (having/not having), social class (according to the classification proposed by the Spanish Society of Epidemiology), physical activity (low/moderate-intense), smoking onset age (before/after 14 years), smoking cumulative consumption (≥10/<10 pack-years), nicotine dependence level (low-moderate or high), motivation to quit smoking level (low-moderate or high), previous attempts to quit smoking (yes/no) and stage of change (pre-contemplation, contemplation or preparation-action ). Potentially confounding variables were excluded from the analysis model. Other variables considered but not included in the final model were: primary healthcare professional performing the intervention, primary healthcare centre, comorbidity disease, body mass index, alcohol intake categorisation, baseline expired-carbon monoxide values, acceptance of a smoking cessation medical visit, intensive motivational intervention, and/or use of pharmacological treatment. | | | |
