# Supplementary material for: Effectiveness of the spirometry-based motivational intervention to quit smoking: RESET randomised trial
Source: Eur J Gen Pract. 2023 Nov 7;29(1):2276764. doi: 10.1080/13814788.2023.2276764 (PMC10631381; doi:10.1080/13814788.2023.2276764)
Supplement: Supplemental Material [file IGEN_A_2276764_SM1523.docx]

Supplementary Table 1. Characteristics of the participants at baseline, according to the randomised assignment group

|  | Control  *n* = 306 | | Intervention  *n* = 308 | | | | |
| --- | --- | --- | --- | --- | --- | --- | --- |
| Baseline sociodemographic variables per study group | | | | |  | | |
| Civil status |  | |  | |  | | |
| - Married | 204 (69.9) | | 218 (71.9)  10 (3.3)  35 (11.6)  40 (13.2) | | | |  |
| - Widower | 9 (3.1) | |  |  |  |  |  |
| - Single | 35 (12.0) | |  |  |  |  |  |
| - Divorced | 44 (15.0) | |  |  |  |  |  |
| Children (number) | 1.73 (1.3) | | 1.57 (1.5) | | | | |
| Educational level |  | |  | |  | | |
| - Illiterate | 5 (1.7) | | 0 (0) | | | | |
| - No schooling but can read and write | 6 (2.1) | | 4 (1.3) | | | | |
| - Unfinished primary education | 28 (9.7) | | 37 (12.3) | | | | |
| - Completed primary education | 143 (49.3) | | 136 (45.3) | | | | |
| - Secondary education (BUP, COU...) | 74 (25.5) | | 88 (29.4) | | | | |
| - Higher education (First cycle) | 16 (5.5) | | 20 (6.7) | | | | |
| - Higher education (Second cycle) | 17 (5.9) | | 15 (5) | | | | |
| - Other | 1 (0.3) | | 0 (0) | | | | |
| Occupation |  | |  | |  | | |
| - Working | 172 (58.1) | | 172 (56.8) | | | | |
| - Unemployed | 60 (20.3) | | 54 (17.8) | | | | |
| - Disability | 11 (3.7) | | 17 (5.6) | | | | |
| - Retired | 28 (9.5) | | 31 (10.2) | | | | |
| - Housewife | 19 (6.4) | | 23 (7.6) | | | | |
| - Student | 2 (0.7) | | 1 (0.3) | | | | |
| - Others | 4 (1.3) | | 5 (1.7) | | | | |
| Social Class ^a^ |  | |  | |  | | |
| - Class I | 13 (4.5) | | 12 (4) | | | | |
| - Class II | 27 (9.3) | | 33 (11) | | | | |
| - Class III | 65 (22.4) | | 64 (21.4) | | | | |
| - Class IVa | 67 (23.1) | | 66 (22.1)  4 8 (16.1) | | | | |
| - Class IVb | 42 (14.4) | |  |  |  |  |  |
| - Class V | 57 (19.7) | | 62 (20.7)  14 (4.7) | | | | |
| - Others | 19 (6.6) | |  |  |  |  |  |
| Medical history | |  | |  | |  |  |
| - Hypertension | | 81 (26.5) | | 85 (27.6) | | |  |
| - Dyslipidaemia | | 74 (24.2) | | 96 (31.2) | | |  |
| - Diabetes mellitus | | 29 (9.5) | | 36 (11.7) | | |  |
| - Coronary disease | | 6 (2) | | 4 (1.3) | | |  |
| - Heart failure | | 3 (1) | | 1 (0.3) | | |  |
| - Other cardiac diseases | | 4 (1.3) | | 1 (0.3) | | |  |
| - Nephro-urological disease | | 0 (0) | | 2 (0.6) | | |  |
| - Neurological disease | | 4 (1.3) | | 4 (1.3) | | |  |
| - Liver disease | | 7 (2.3) | | 6 (1.9) | | |  |
| - Gastrointestinal disease | | 9 (2.9) | | 9 (2.9) | | |  |
| - Psychiatric disease | | 45 (14.7) | | 56 (18.2) | | |  |
| - Rheumatological disease | | 20 (6.5) | | 24 (7.8) | | |  |
| Health habits   - Physical activity  1. Light (office worker, health worker) 2. Moderate (industry, students) 3. High (construction workers, forest ranger) | | 148 (50)  134 (45.3)  14 (4.7) | | 139 (46.2)  138 (45.8)  24 (8) | | |  |
| - Physical activity time (minutes/week) | | 183 (12.8) | | 182 (13.8) | | |  |
| - Alcohol intake ^b^ | |  | |  | |  |  |
| 1. Low Risk | | 281 (94.3) | | 278 (91.7) | | |  |
| 1. High Risk | | 17 (5.7) | | 25 (8,3) | | |  |
| - Alcohol intake (SD/week) | | 5.07 (0.6) | | 5.96 (0.6) | | |  |
| Physical exam | |  | |  | |  |  |
| - Height (cm) | | 165.21 (8.5) | | 164.46 (10.7) | | |  |
| - Weight (Kg) | | 74.98 (2.4) | | 74.63 (0.9) | | |  |
| - BMI (Kg/m^2^) | | 27.33 (14.0) | | 27.84 (8.9) | | |  |
| - Waist circumference (cm) | | 93.08 (13,8) | | 94.47 (14.9) | | |  |
| - Anthropometric categorisation ^c^ | |  | |  | |  |  |
| 1. Underweight | | 3 (1.0) | | 5 (1.6) | | |  |
| 1. Healthy weight | | 122 (40.8) | | 106 (34.6) | | |  |
| 1. Class I overweight | | 47 (15.7) | | 47 (15.3) | | |  |
| 1. Class II overweight | | 61 (20.4) | | 55 (17.9) | | |  |
| 1. Class I Obesity | | 52 (17.5) | | 67 (21.8) | | |  |
| 1. Class II Obesity | | 9 (3.0) | | 21 (6.8) | | |  |
| 1. Class III Obesity | | 4 (1.3) | | 5 (1.7) | | |  |
| 1. Class IV Obesity | | 1 (0.3) | | 1 (0.3) | | |  |
| - Systolic blood pressure (mmHg) | | 124.3 (1) | | 125.6 (0.9) | | |  |
| - Diastolic blood pressure (mmHg) | | 75.4 (0.6) | | 76.2 (0.6) | | |  |
| - Oxygen saturation (%) | | 97.3 (0.4) | | 97.3 (0.3) | | |  |
| Data are presented as number of patients (and percentage) or mean (and standard deviation) according to the type of variable.  SD: Standard Drink; BMI: Body Mass Index  (a) According to the classification proposed by the Spanish Society of Epidemiology (Domingo-Salvany 2000)  (b) Consumption equal or over 28 SD/ week in men or 17 SD/ week in women is considered risk.  (c) According to the Spanish Society for the Study of Obesity 2016 | | | | | | |  |
