# Supplementary material for: Effectiveness of the spirometry-based motivational intervention to quit smoking: RESET randomised trial
Source: Eur J Gen Pract. 2023 Nov 7;29(1):2276764. doi: 10.1080/13814788.2023.2276764 (PMC10631381; doi:10.1080/13814788.2023.2276764)
Supplement: Supplemental Material [file IGEN_A_2276764_SM1521.docx]

Supplementary File 1. RESET study researchers list

**CAP Bonavista**: D Borras-Vicente, F Gomez-Santidrian, M Grive-Isern, D Jovani-Puig, MT Juncosa-Cabré, MT Martinez-Perez, N Sarra-Manetas; **CAP Constanti**: E Aragones-Benaiges, S Exposito-Ribau, E Ferrer-Sorribas, S Folch-Pujol, M Fores-Palacios, P Perez-Layarra; **CAP Cornudella del Montsant:** F Buj-Visiedo, I Gallego-Arteaga, N Guinjoan-Aymemi, A Poca-Pastor, M Vila-Molet; **CAP El Morell:** MT Canela-Armengol , J Daniel-Diez, I Farre-Torra, M Garcia-Olive, MD Gil-Sanchez, C Senosiain-Yerno, R Vila-Ruiz; **CAP Falset**: MJ Andres-Pablo, JM Hernandez-Anguera, AM Lara-Pedrosa, T Lara-Pedrosa, M Lluís-Burgueño, M Mengual-Miralles L Pelleja-Pellicer, R Rodriguez-Perez, M Rodríguez-Miguélez, M Sabate-Margalef, R Subirats-Segarra; **CAP Flix**: N Allende-Muntane, T Aviño-Llopis, M Boira-Costa, C Delgado-Azuara, M Garcia-Vaque, S Loran-Valcarcel, T Sangra-Rodes; **CAP Horts de Miro:** E Alvarez-Soler, MT Basora-Gallisa, J Blade-Creixenti, J Breva-Aymerich, A Caballero-Alias, A DelPozo-Niubo, N Martin-Vergara, A Vinuesa-Fernandez; **CAP Jaume I**: MG Aguirre-Alava, S Crispi-Cifuentes, Y Fernandez-Pages, A Garcia-Uriarte, R Solis-Narvaez; **CAP Llibertat**: R Caro-Garduño, C Cortes-Ponce, C Ferrer-Marin, T Llauradó-Sabaté, M Llauradó-Vernet, P Llobet-Azpitarte, C Mangrane-Guillen, G Muñoz-Alvarez, MA Oliver-Esteve, F París-Pallejà, M Ricart-Sancho, E Rivera-Manrique, A Salva-Brusel, M Soler-Pont, M Timon-Torres; **CAP Montblanc**: M Boldú-Ortega, G Elizalde-Río, C GarciaTalarn, M Gorgues-Espasa, A Güell-Coll, C Hernandez-Nuñez, MA Naranjo-Orihuela, A Odena-Estrade, I Palou-Vall, L Panadès-Baldrich, A Ribé-Miró; **CAP Salou**: Y Ortega-Vila, I Pascual-Palacios, X Perez-Cuit, M Rodrigo-Gotor; **CAP Sant Pere**: M Aliseda-Tienza, C Anguera-Perpiña, J Boj-Casajuana, JJ Cabré-Vila, C Chancho-Rodriguez, S Dalmau-Vidal, M Español-Pons, M García-Barco, R Gonzalez-Perez, M Huguet-Jacob, A Isach- Subirana, E Martí-Suau, M Munté-Bigorra, M Nolla-Mallafre, J Pardo-Andújar, R Pedret-Llaberia, L Peralta-Encinas, P Preixens-Vallinoto, E Ras-Vidal, C Rubio-Gascon, A Reche-Martinez, R Sagarra-Alamo, M Sanchez-Marin, MI Sanchez-Oro, AR Silva-Orjuela, J Vizcaíno-Marin; **CAP Sant Pau**: L Augé-Garcia, AI Castelao-Alvarez; **CAP Sant Salvador**: F Calamote-Manso, L Clotas-Sancho, J Ferre-Gras, M Gasull-Gomis, A Gonzalez-Garces, S Gonzalez-Gonzalo, A Llano-Sanchez, E Nevot-Bueno, E Ruiz-Morcillo; **CAP Tarraco**: MS delAmo-Benito, E Guijarro-Tapia, AJ Lopez-Barea, A Manresa-Font, I Marsà-Gimènez, R Moreno-Ramon, N Nievas-Diaz, M Prieto-Cid, M Pujol-Porquera, C Toledo-Peinado; **CAP Torreforta**: ML Barrera-Uriarte, EJ Borreguero-Guerrero, MP Castell-Montrull, RE Catalin, P CentellesVelilla, J Ferre-Rey, MJ Forcadell-Peris, CM Fuentes-Bellido, O Garcia-Gimeno, R Landin-Delgado, J Ledo-Garcia, E Satue-Gracia; **CAP Valls**: O Briones-Carcedo, C Rambla-Vidal, J Robuste-Ingles; **CAP Vilarodona**: OE Arago-Albalate, VR Arnau-Adan, EN Balcells-Gonzalez, A Boada-Tous, G Cando-Guasch, J Casajuana-Brunet, J Castellvi-Baldira, R Girona-Real, MC Grau-Perez, M Medina-Clemente, A Moreno-Lagunas, MC Moreno-Ortiz, E Pay-Caro, AM Prats-Caellas, R Profitos-Amiell, F Rodriguez-Alonso, FJ Sobrino-Perez, P Sole-Barreras, ML Villaroya- Bullido.
